# EEMD-MUSIC-Based Analysis for Natural Frequencies Identification of Structures Using Artificial and Natural Excitations

**DOI:** 10.1155/2014/587671

**Published:** 2014-02-10

**Authors:** David Camarena-Martinez, Juan P. Amezquita-Sanchez, Martin Valtierra-Rodriguez, Rene J. Romero-Troncoso, Roque A. Osornio-Rios, Arturo Garcia-Perez

**Affiliations:** ^1^HSPdigital-CA Mecatronica, Facultad de Ingenieria, Universidad Autonoma de Queretaro, Campus San Juan del Rio, Rio Moctezuma 249, 76807 San Juan del Rio, QRO, Mexico; ^2^HSPdigital-CA Telematica, Procesamiento Digital de Señales, DICIS, Universidad de Guanajuato, Carr. Salamanca-Valle km 3.5+1.8, Palo Blanco, 36700 Salamanca, GTO, Mexico

## Abstract

This paper presents a new EEMD-MUSIC- (ensemble empirical mode decomposition-multiple signal classification-) based methodology to identify modal frequencies in structures ranging from free and ambient vibration signals produced by artificial and natural excitations and also considering several factors as nonstationary effects, close modal frequencies, and noisy environments, which are common situations where several techniques reported in literature fail. The EEMD and MUSIC methods are used to decompose the vibration signal into a set of IMFs (intrinsic mode functions) and to identify the natural frequencies of a structure, respectively. The effectiveness of the proposed methodology has been validated and tested with synthetic signals and under real operating conditions. The experiments are focused on extracting the natural frequencies of a truss-type scaled structure and of a bridge used for both highway traffic and pedestrians. Results show the proposed methodology as a suitable solution for natural frequencies identification of structures from free and ambient vibration signals.

## 1. Introduction

Nowadays, the vibration-based structural health monitoring (SHM) is a major and fast growing research discipline for several fields such as mechanical engineering, aeronautics, and civil engineering, among others, because it allows the examination of the dynamic characteristics of a specific structure. The basic idea in vibration-based SHM is that physical variations in the structure change its modal parameters and, consequently, its vibration response also experiments disturbances. Therefore, accurate identification and measurement of modal parameters such as natural frequencies are fundamental in vibration-based SHM in order to estimate different structural conditions [1, 2]. Moreover, a correct identification of modal parameters allows building a proper analytical model, as well as determining the existence and location of structural damage, and in some cases calculating the lifetime of the structure [3]. Several vibration excitation sources have been used to excite civil structures in order to measure their modal properties, which can be classified into two groups. The first one named artificial excitation uses mechanical shakers, drop weights, shoot rockets, control devices, and so on. The second group, named natural excitation, uses ambient vibrations such as wind and earthquakes. The ambient vibration testing has the advantage of being inexpensive and not interrupting the normal operation since no excitation equipment and traffic interruption are needed and also has potentials for implementing real-time condition assessment [4–6]. However, the modal parameter identification of civil engineering structures using the field ambient vibration measurement is a complicated procedure, since ambient vibration data is nonstationary and it is embedded in high-level noise. Therefore, it is desirable to have a reliable data analysis or signal processing method capable of analyzing ambient vibrations.

In the past few years, a number of different techniques have been proposed to estimate the modal parameters of a structure such as the peak-picking method (PP) [7], the frequency response function (FRF) [8], the natural excitation technique (NexT) [9], the stochastic subspace identification method (SSI) [10, 11], the multivariate AR model [12], the autoregressive-moving average model (ARMA) [13], the eigensystem realization algorithm (ERA) [14], the enhanced frequency domain decomposition (EFDD) [15], and the McKelvey frequency domain subspace algorithm [14]. However, these methods assume the structural response is stationary in order to simplify the signal processing, which is not true in many cases, and therefore the modal parameters cannot be adequately analyzed by these methods [4]. Besides, some methods need a large set of data to identify accurately the modal parameters and, moreover, the results may be affected in noisy conditions [16, 17]. To overcome these drawbacks, new tools, such as the wavelet transform (WT) [18, 19], the Hilbert-Huang transform (HHT) [20–22], and the empirical mode decomposition (EMD) with random decrement (RD) [4], have been used to estimate the modal parameters of a structure as well as for damage detection. The WT method provides the time-scale analysis of a transient and nonstationary signal since it decomposes the signal into time-scale representation rather than time-frequency representation. Unfortunately, the WT capabilities are significantly degraded in noisy environments, as the case of real applications; besides, it requires many decomposition levels to be able to measure the modal parameters [19]. The HHT provides a time-frequency representation of nonstationary and transient signals. Its characteristics have been demonstrated to be more precise for decomposing a signal in the time-frequency domain than WT [23]. The HHT is based on two-steps, which are the EMD and the Hilbert transform (HT) methodologies. The EMD method decomposes any data into a set of band-limited quasistationary functions, called intrinsic mode functions (IMFs); later, the Hilbert transform is applied to each IMF, which allows obtaining the amplitude and phase angle of each IMF. Regrettably, the major drawback of EMD method is the so-called mode mixing effect, where the mode mixing indicates that oscillations with the same time scale have been assigned to different IMFs [24]. For this reason, [25] proposed the ensemble EMD (EEMD) method in order to solve the problems of mode mixing caused by an intermittent frequency in the EMD method. Despite this, the EEMD-HT, EMD-HT, and EMD-RD methods have some difficulties to detect or extract close space modes, which are present in many civil structures due to the symmetrical geometry and similar physical properties in different directions. In addition, these methods are also very susceptible to noise. Therefore, it would be desirable to have a technique that helps the EEMD to extract the natural frequencies of a structure with great efficiency and accuracy, especially those modes that are very close. Regarding close frequency components, a high-resolution spectral estimation through the multiple signal classification (MUSIC) algorithm has been successfully presented for very close frequency components, even for data with low signal-to-noise ratio [26, 27]; therefore, the MUSIC algorithm is a promising technique to detect the close space modes of the structures.

The contribution of this paper is to present a new EEMD-MUSIC-based methodology to identify modal frequencies in structures ranging from free and ambient vibration signals produced by artificial and natural excitations. The EEMD is used to decompose the vibration signal into a set of IMFs and then MUSIC identifies the natural frequencies of the structure. The usefulness and effectiveness of the proposed methodology are assessed through three experimental cases. In the first experiment, a numerical simulation is carried out to validate the accuracy and noise immunity of the proposed methodology for extracting the natural frequencies, especially when the modal frequencies are close. The second experiment is focused on extracting the modes of a truss-type scaled structure by using an artificial excitation, where the results are compared with the obtained values through finite element analysis (FEA). Finally, the third experiment concentrates on identifying the natural frequencies of a bridge with natural excitation by using a set of field ambient vibration measurements. Analytical and experimental results show an excellent identification of the natural modes even when the signal is embedded in high-level noise, and, more importantly, the results demonstrate that the methodology provides a suitable alternative approach to natural frequencies identification from free and ambient data by using artificial and natural excitations.

## 2. Mathematical Background

This section presents the mathematical background of the proposed techniques for vibration analysis of structures.

### 2.1. Empirical Mode Decomposition

The EMD method is a data-processing technique for analyzing nonstationary and nonlinear signals. The EMD method decomposes the vibration signal into a set of band-limited quasistationary functions called IMFs. Each IMF has to satisfy the following two conditions: (i) the number of zero crossings and the number of extrema must either equal or differ by at most one and (ii) the mean value of the envelope defined by the local maxima and the envelope defined by the local minima must be zero [28].

The process for obtaining each IMF is called the sifting process, which is described as follows.

(1) Identify the maxima and minima of the signal *x*(*t*).
(2) Generate an upper and lower envelop by using cubic spline interpolation. The average of the two envelops is *m*_1_(*t*). Subtract *m*_1_(*t*) from the original signal *x*(*t*) to obtain
  (1) $$\begin{array}{c} h_{1}\left(t\right)=x\left(t\right)-m_{1}\left(t\right). \end{array}$$
  Determine if (1) satisfies the conditions (i) and (ii). If not, repeat the first two steps until *h*_*k*_(*t*) satisfies the conditions (i) and (ii); then, *h*_*k*_(*t*) is the first IMF defined as
  (2) $$\begin{array}{c} c_{1}\left(t\right)=h_{k}\left(t\right)=\text{IMF}1.\; \end{array}$$
(3) After IMF1 is obtained, subtract *c*_1_ from the original signal *x*(*t*)and calculate the residue signal as follows:
  (3) $$\begin{array}{c} r_{1}\left(t\right)=x\left(t\right)-c_{1}\left(t\right). \end{array}$$
(4) Treat *r*_1_ as the original signal and repeat the procedure from step (1) to step (3) to obtain the other IMF (*c*_2_, *c*_3_,…, *c*_*n*_). The process is stopped when the final residual signal *r*_*n*_(*t*) is a monotonic function.
(5) At the end of the procedure, the signal *x*(*t*) is decomposed into *n* intrinsic modes *c*_*i*_(*t*) and a residue *r*_*n*_(*t*). Now, the original signal can be represented by
  (4) $$\begin{array}{c} x\left(t\right)=\sum_{i=1}^{n}c_{i}\left(t\right)+r_{n}\left(t\right). \end{array}$$

### 2.2. Ensemble Empirical Mode Decomposition

The EEMD is an adaptive and noise-assisted method proposed by [25, 29] in order to improve the shortcomings of the EMD algorithm and provide a feasible solution of mode mixing caused by an intermittent frequency. The EEMD method defines the IMF set for an ensemble of trials, each one obtained by applying the EMD to the signal of interest with added independent identically distributed white noise of the same standard deviation.

According to [25], EEMD contains the following steps.

1. Add a white noise series to the targeted data.
2. Decompose the data with added white noise by EMD algorithm, described in Section 2.1.
3. Repeat steps (1) and (2) until the trial number is met, but with different white noise series each time.
4. Estimate the (ensemble) means of corresponding IMF of the decompositions as the desired output.

### 2.3. MUSIC Algorithm

MUSIC algorithm is known as a high-resolution method which can detect frequencies with low signal-to-noise ratio. MUSIC assumes that the discrete time signal *x*[*n*] can be represented as a sum of *m* complex sinusoid in noise *e*[*n*] [30]. Consider

(5) $$\begin{array}{c} x\left[n\right]=\sum_{i=1}^{m}\overset{-}{B_{i}}e^{j2\pi f_{i}n}+e\left[n\right],\;n=0,1,2,\ldots ,N-1 \end{array}$$

with

(6) $$\begin{array}{c} \overset{-}{B_{i}}=\left|B_{i}\right|e^{\phi_{i}}, \end{array}$$

where *N*, *B*_*i*_, and*f*_*i*_ are the number of sample data, the complex amplitude, and its frequency, respectively, and *e*[*n*] is a sequence of white noise with zero mean and a variance *σ* ^2^. This method uses the eigenvector decomposition of *x*[*n*] to obtain two orthogonal subspaces. The autocorrelation matrix *R* of the noisy signal *x*[*n*] is the sum of the autocorrelation matrices of the pure signal *R*_*s*_ and the noisy *R*_*n*_ as follows:

(7) $$\begin{array}{c} R=R_{s}+R_{n}=\sum_{i=1}^{P}{\left|B_{i}\right|}^{2}e\left(f_{i}\right)e^{H}\left(f_{i}\right)+\sigma_{n}^{2}I, \end{array}$$

where *P* is the number of frequencies, the exponent *H* denotes Hermitian transpose, *I* is the identity matrix, and *e* ^*H*^(*f*_*i*_) is the signal vector given by

(8) $$\begin{array}{c} e^{H}\left(f_{i}\right)=\left\lfloor \begin{array}{cccc} 1 & e^{-j2\pi f_{i}} & \cdots & e^{-j2\pi f_{i}(N-1)} \end{array}\right\rfloor . \end{array}$$

From the orthogonality condition of both subspaces, the MUSIC pseudospectrum *Q* of the current space vector is given by

(9) $$\begin{array}{c} Q^{\text{MUSIC}}\left(f\right)=\frac{1}{{\left|e{\left(f\right)}^{H}V_{m+1}\right|}^{2}}, \end{array}$$

where *V*_*m*+1_ is the noise eigenvector. This expression exhibits the peaks that are exactly at frequency of principal sinusoidal component, where *e*(*f*)^*H*^ *V*_*m*+1_ = 0.

## 3. Proposed Methodology

**Figure 1:**
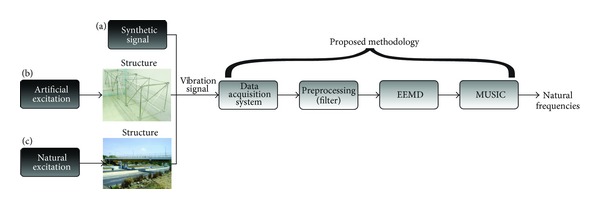
Proposed methodology. (a) Synthetic signal, (b) artificial excitation, and (c) natural excitation.

Figure 1 shows the flowchart of the proposed EEMD-MUSIC-based methodology for identifying modal frequencies from free and ambient vibration signals produced by an artificial or a natural excitation. The first step is to acquire the vibration signal of the structure. Second, the sampled vibration signal is passed through a band-pass filter, in order to extract the bandwidth of interest related to the modal frequencies as well as to help solve the mode mixing. Third, the filtered vibration signal is decomposed into its IMF utilizing the EEMD. Finally, the MUSIC algorithm is applied to each IMF to compute the modal frequencies of the structure.

The usefulness of the proposed methodology is tested in three experiments. The first experiment focuses on the use of a synthetic signal to validate the accuracy and noise immunity of the proposed methodology (Figure 1(a)). In the second experiment, the proposed methodology is tested on a truss-type scaled structure located in a laboratory, where the excitation conditions are controlled (Figure 1(b)). Finally, the third experiment is carried out to show the effectiveness of the proposed methodology under real operating conditions. In this experiment, a real structure (a bridge) is analyzed, in which the natural excitation is used (Figure 1(c)).

## 4. Experimentation and Results

In order to show the effectiveness of the proposed methodology, three experiments are performed. The first experiment consists of a numerical simulation implemented to validate the accuracy and noise immunity of the proposed methodology for extracting the natural frequencies, especially when the natural frequencies are close. Further, the proposed methodology and different approaches are compared in order to validate their efficiency by estimating the natural frequencies. The second experiment concentrates on an artificial excitation of the free decay under forced vibration that is applied on a truss-type scaled structure for extracting the natural frequencies. The experimental results are compared with those obtained by FEA. Finally, the third experiment concentrates on identifying the natural frequencies of a bridge with natural excitation by using a set of field ambient vibration measurements. This experimentation takes into account that ambient vibration data is nonstationary and it is embedded in high-level noise.

### 4.1. Synthetic Signal

**Figure 2:**
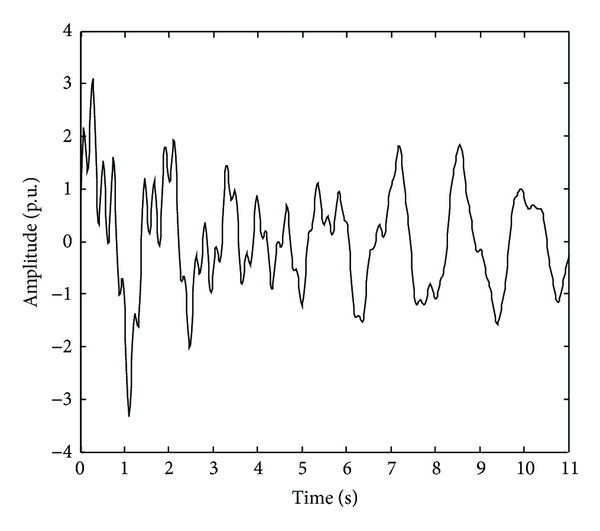
Synthetic signal.

In order to validate the proposed methodology performance, a comparison between different approaches, EEMD-FFT and -HT, and the discrete wavelet transform (DWT)-FFT, -HT, and -MUSIC methods is presented. For this, a synthetic signal with *a priori* known values is used, which appears in Figure 2 and corresponds to a damped free vibration response of 4-degree of freedom system as follows:

(10) $$\begin{array}{c} i\left(t\right)=\sum_{j=1}^{4}A_{j}e^{-2\pi \zeta_{j}f_{j}t}\sin\left(2\pi fd_{j}t+\theta_{j}\right)+\omega \left(t\right), \end{array}$$

where *Aj*, *θj*, *fj*, $fd=f_{j}\sqrt{1-\zeta_{j}^{2}}$, and *ζj*are the amplitude, phase, undamped natural frequency, damped natural frequency, and damping ratio of the *j*th mode, respectively, whereas *ω*(*t*) is a sequence of white noise. The used numerical values are *f*_1_ = 0.61 Hz, *f*_2_ = 0.73 Hz, *f*_3_ = 1.56 Hz, *f*_4_ = 4.35 Hz, *ζ*_*j*_ = 0.01, *A*_*j*_ = 1.0, and *θ*_*j*_ = 0, for *j* = 1, 2, 3, and 4, with a sampling frequency and sampling time of 50 Hz and 11 s, respectively. Besides, a 30% level noise for an SNR = 7.4 dB is used to test the efficiency and immunity of the techniques to noisy environments.

**Figure 3:**
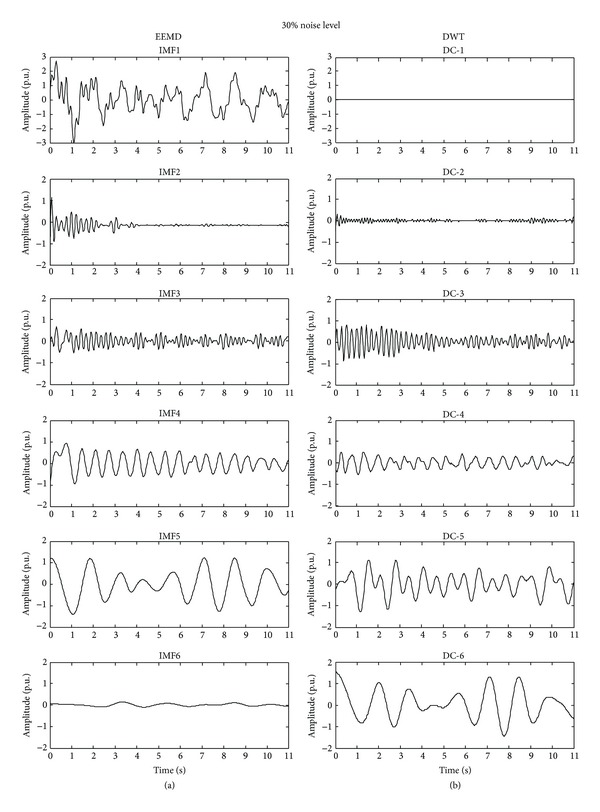
IMFs and DCs obtained of the synthetic signal through EEMD and DWT, respectively.

Firstly, the synthetic signal is decomposed, as shown in Figure 3, by the EEMD and DWT which is also a technique to decompose a signal into frequency bands named decompositions (DCs) [3]. Then, some IMFs and DCs are chosen for being analyzed. Regarding the IMFs (Figure 3(a)), the first two IMF levels are discarded since they contain the noise and high-frequency components; therefore, only the IMF3, IMF4, and IMF5 are considered to identify the natural modes. On the other hand and according to the DCs (Figure 3(b)), the first two decompositions, DC_1_ and DC_2_, are discarded since they contain the high-frequency bands; consequently, the DC_3 _(3.125–6.25 Hz), DC_5_ (0.781–1.5625 Hz), and DC_6_ (0.390–0.781 Hz) are analyzed to identify the natural frequencies since they contain the frequencies of interest.

**Figure 4:**
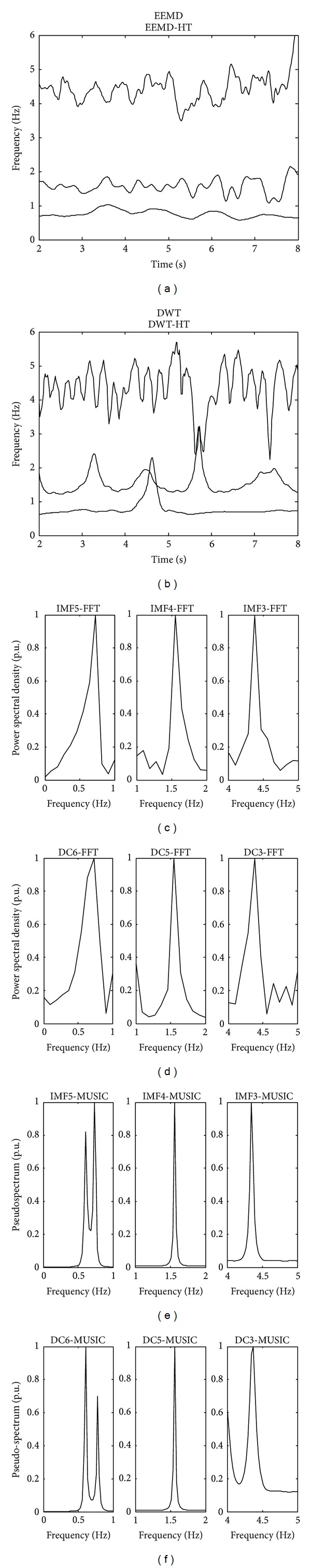
Results obtained by (a) EEMD-HT method, (b) DWT-HT, (c) EEMD-FFT, (d) DWT-FFT, (e) EEMD-MUSIC, and (f) DWT-MUSIC.

Later, the selected IMFs and DCs are processed by the HT, the FFT, and the proposed MUSIC method as shown in Figure 4. From these figures, it is possible to observe that the EEMD-HT method (Figure 4(a)) and the DWT-HT method (Figure 4(b)) only detect three modes due to the fact that (i) the EEMD and DWT cannot separate close frequencies and (ii) that the HT can only identify one frequency by each IMF and DC, respectively. Similarly, the EEMD-FFT (Figure 4(c)) and the DWT-FFT (Figure 4(d)) cannot identify close modes due to overlapping frequency components and to the FFT resolution. On the other hand, the EEMD-MUSIC (Figure 4(e)) and the DWT-MUSIC (Figure 4(f)) identify clearly the four natural modes; however, the results for the natural frequencies obtained by DWT-MUSIC are degraded in noisy environments unlike the EEMD-MUSIC method. All the aforementioned results are presented in a normalized way for a better presentation and understanding.

Finally, Table 1 shows the numerical comparison between the reference values of the synthetic signal and the obtained values through the EEMD-HT, the EEMD-FFT, the DWT-HT, the DWT-FFT, the DWT-MUSIC, and the proposed EEMD-MUSIC methods. As a result of the capabilities of the EEMD-HT, the DWT-HT, the EEMD-FFT, and the DWT-FFT methods, the closest natural modes of the synthetic signal, *f*_1_ and *f*_2_, are mixed and, therefore, only one value of them is obtained. On the other hand, the proposed EEMD-MUSIC method proved to be able to extract the four natural modes with higher accuracy than DWT-MUSIC since the results are affected by the noise level.

The overall proposed methodology for this and the two next study cases is implemented in the MATLAB Digital Signal Processing Toolbox. The MUSIC algorithm has an order of 4 and overlaps of 25%. Regarding the EEMD, the number of ensemble members is set to 100 and the standard deviation of white-noise series is set to 0.1. On the other hand, the Daubechies (db10) window is used as the mother wavelet in the DWT.

### 4.2. Artificial Excitation

**Figure 5:**
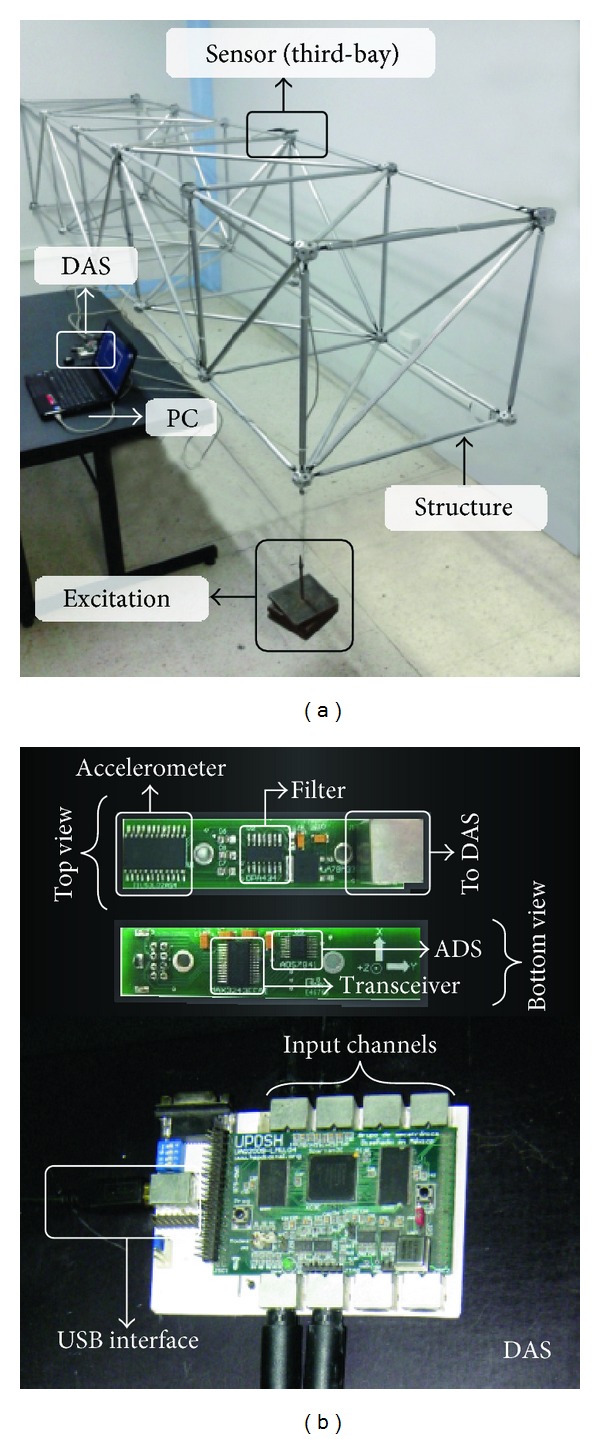
Experimental setup: (a) overall system and (b) triaxial accelerometer and data acquisition system.

**Figure 6:**
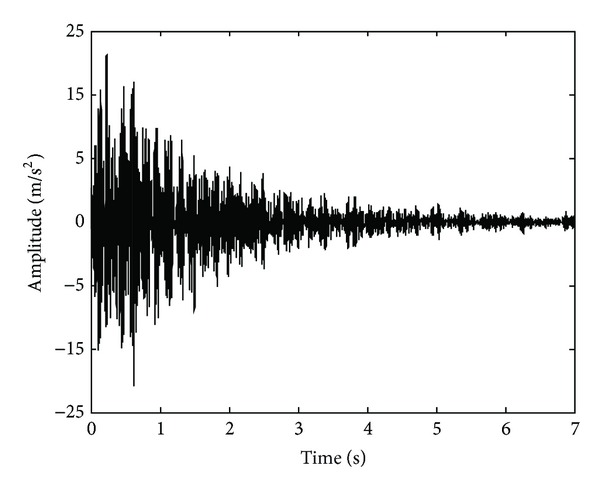
Measured vertical acceleration.

In the second experiment, a tridimensional truss-type scaled structure, shown in Figure 5(a), is used for extracting the natural frequencies by means of an artificial excitation. The modal testing is performed using a hanging mass of 14 kg as excitation source. The mass is tied in the first-bay in order to have the maximum displacement of the structure; then, the wire that subjects the mass is cut to induce a free vibration in the structure. The vibration signal is acquired using a MEMS-based triaxial accelerometer model LIS3L02AS4 from STMicroelectronics placed on top of the third bay as shown in Figure 5(a). The accelerometer has a user-selectable full scale of ± 19.6 m/s^2^/± 58.8 m/s^2^ with a 50 × 10^−4^ m/s^2^ resolution over 100 Hz. The accelerometer information is digitalized using a 12-bit 4-channel ADS7841 analog-to-digital converter (ADC) shown in Figure 5(b) from Texas Instruments, with a maximum sampling rate of 200 kHz in each channel. The obtained signals from the accelerometer are stored in a proprietary data acquisition system (DAS) and sent to a personal computer (PC) by universal serial bus (USB) protocol as shown in Figure 5(b). The DAS uses a sampling frequency of 3.2 kHz for obtaining 22,400 samples during a time window of 7 s. The vertical free vibration signal, shown in Figure 6, is used to identify the vertical natural frequencies of the structure.

**Figure 7:**
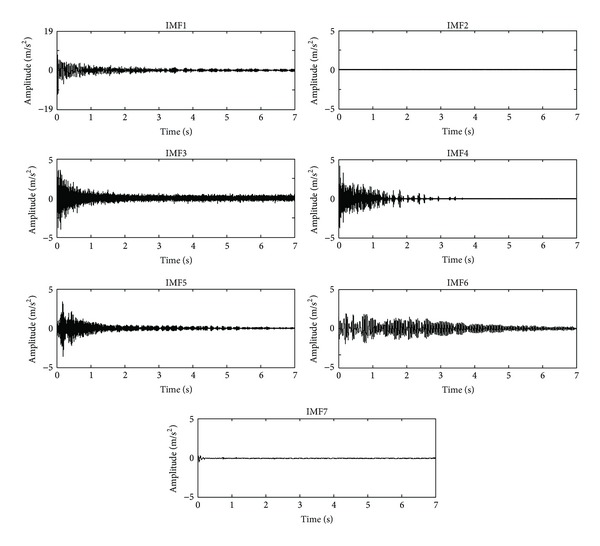
IMF obtained through EEMD for free vibration acceleration.

**Figure 8:**
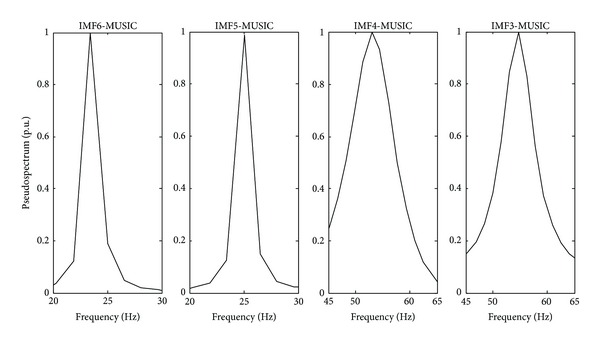
Pseudospectra of different analyzed IMF.

Figure 7 shows the first seven IMFs obtained from the free vibration response signals. The IMF1 and IMF2 contain noise and high-frequency components out of the frequency range of interest. On the other hand, from IMF3 to IMF6 are used for analysis with the MUSIC method because they contain the natural frequencies for the truss-type structure. The rest of the IMFs are discarded since they do not contain relevant information.  Figure 8 displays the normalized pseudospectrum obtained from selected IMF, where each one of the natural frequencies is observed. The identification results by the proposed methodology are compared to those from FEA as well as from the experimental result identified with the DWT-MUSIC method. The obtained results are presented in Table 2. It is worth noticing that only the DWT-MUSIC is used as an experimental comparative technique since it has the second best performance after EEMD-MUSIC, as shown in the previous study case. The proposed methodology identifies the natural frequencies reasonably closed when it is compared with the analytical estimation from the FEA. Evidently the small differences may be due to a number of real conditions that are not considered or completely represented in the FEA. On the contrary, the DWT-MUSIC method has a higher error in the identification of the natural frequencies due to the real noisy environment.

### 4.3. Natural Excitation

**Figure 9:**
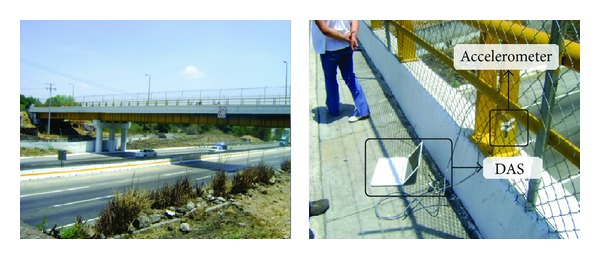
Analyzed bridge.

**Figure 10:**
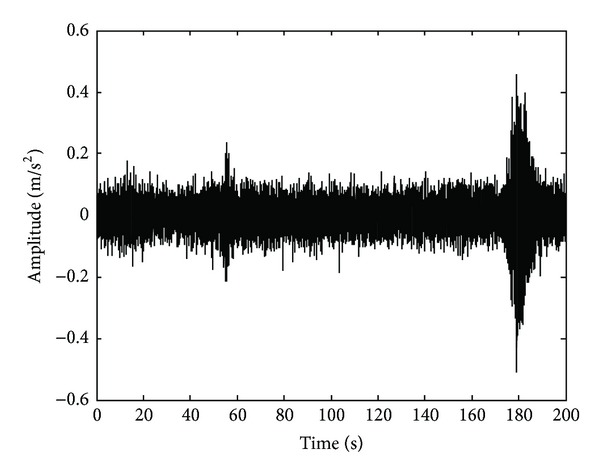
Measured acceleration response.

**Figure 11:**
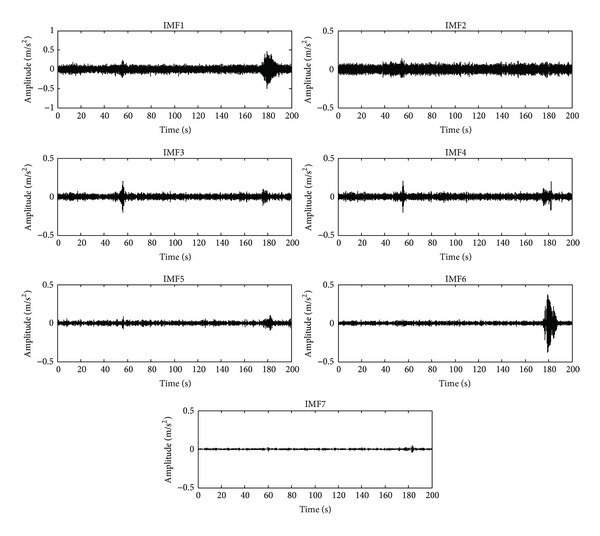
IMFs obtained through EEMD for ambient vibration acceleration.

**Figure 12:**
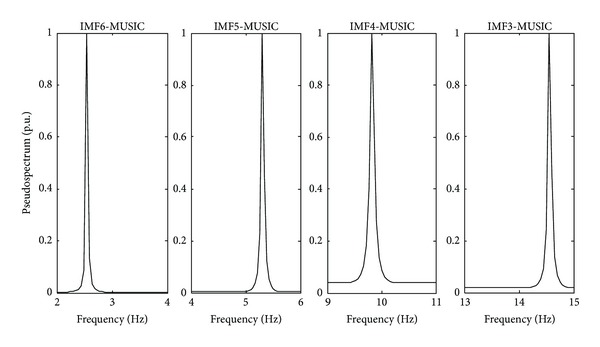
Pseudospectra of different analyzed IMFs.

The bridge considered in this study is located in “San Juan del Rio, Queretaro, Mexico.” The bridge is used for both highway traffic and pedestrians as shown in Figure 9. The ambient vibration caused by traffic is on-line monitored through a triaxial accelerometer mounted in the middle of the bridge. The obtained signals from the accelerometer are stored in the DAS and sent to PC by USB protocol. The DAS uses a sampling frequency of 160 Hz for obtaining 32,000 samples during a time window of 200 s, where the vertical vibration response is plotted in Figure 10, and the filtered signal is analyzed through the EEMD method, and its results are shown in Figure 11. The IMF3 to IMF6 are selected to obtain the natural frequencies since it contains the interested frequency range of the bridge.  Figure 12 displays the normalized pseudospectra obtained from selected IMF, where each one of the natural frequencies is observed (2.539 Hz, 5.313 Hz, 9.814 Hz, and 14.55 Hz).

Table 3 shows a comparison between the values obtained from the proposed methodology and the results obtained by the DWT-MUSIC method. This method can detect the four natural frequencies; however, their accuracy is expected to be lower due to the noise, since ambient vibration data is embedded in high-level noise.

## 5. Conclusions

In this paper, a new methodology based on the fusion of the EEMD and the MUSIC methods for identifying the natural frequencies of civil structures from free and ambient vibration response signals produced by artificial and natural excitations is presented. The effectiveness of the proposal is demonstrated through three experiments. (i) The numerical simulation shows that the proposal can identify closely spaced frequencies even in noisy environments unlike the EEMD-HT, DWT-HT, EEMD-FFT, and DWT-FFT methods; although the DWT-MUSIC method is able to detect close modes, it is sensitive to noisy environments, as the case of free and ambient vibration signals, which does not make it suitable for identifying accurately the natural modes, which is essential in the design of civil structures [31]. Besides, the decomposition level of the DWT has to be known or at least supposed *a priori *in order to have an adequate result, which is very difficult in real applications since all the signals may have different modes; consequently, the frequency band that contains the natural mode is also different. On the other hand, the EEMD does not have this drawback since it is an intuitive, unsupervised, and self-adaptive method that gives an automatic result. (ii) The very similar obtained natural frequencies through the proposal and FEM of a real truss-type structure make the proposal a suitable tool for the analysis of free vibration signals produced by artificial excitations. (iii) EEMD-MUSIC is a powerful approach for identifying natural frequencies in real and non-controlled civil structures (i.e. a bridge) from ambient vibration data, produced by natural excitations. which is a complicated task since they are nonstationary and embedded in high-level noise.

The immunity to noisy environments and the capability to identify closely spaced frequencies with high accuracy make the proposal a suitable and powerful tool for different research disciplines such as SHM, structural damage detection, and design structure, helping in the development of appropriate and accurate analytical models for improving the performance, resistance, and life service of the structures, among others.

## Figures and Tables

**Table 1:** Results of identified natural frequencies with different methodologies.

Reference values	EEMD			DWT		
	HT	FFT	MUSIC	HT	FFT	MUSIC
*f*_1_ = 0.61	0.72*	0.68*	**0.61**	0.70*	0.72*	0.61
*f*_2_ = 0.73			**0.73**			0.78
*f*_3_ = 1.56	1.54	1.52	**1.56**	1.31	1.55	1.56
*f*_4_ = 4.35	4.38	4.30	**4.34**	4.41	4.38	4.37

*Mixed modes.

**Table 2:** Comparison of natural frequencies.

Modes	Finite element analysis	Proposed methodology (EEMD-MUSIC)	DWT-MUSIC
Natural frequency (Hz)			
1	24.48	23.44	21.88
2	25.50	25.00	26.56
3	52.57	51.56	50.00
4	54.65	54.69	53.13

**Table 3:** Identified natural frequencies.

Modes	Proposed methodology	DWT-MUSIC
Natural frequencies (Hz)		
1	2.539	2.50
2	5.313	5.15
3	9.814	10.00
4	14.55	14.69
